# Genomic Organization of Repetitive DNA Elements and Its Implications for the Chromosomal Evolution of Channid Fishes (Actinopterygii, Perciformes)

**DOI:** 10.1371/journal.pone.0130199

**Published:** 2015-06-12

**Authors:** Marcelo de Bello Cioffi, Luiz Antonio Carlos Bertollo, Mateo Andres Villa, Ezequiel Aguiar de Oliveira, Alongklod Tanomtong, Cassia Fernanda Yano, Weerayuth Supiwong, Arunrat Chaveerach

**Affiliations:** 1 Departamento de Genética e Evolução, Universidade Federal de São Carlos, São Carlos, São Paulo, Brazil; 2 Franklin College of Arts and Sciences, University of Georgia, Athens, Georgia, United States of America; 3 Department of Biology, Faculty of Science, Khon Kaen University, Muang District, Khon Kaen, Thailand; 4 Faculty of applied science and engineering, Khon Kaen University, Nong Kai Campus, Muang, Nong Kai, 43000, Thailand; 5 Genetics and Environmental Toxicology Research Group, Khon Kaen University, Muang District, Khon Kaen, Thailand; Institute of Cotton Research of Chinese Academy of Agricultural Sciences, CHINA

## Abstract

Channid fishes, commonly referred to as “snakeheads”, are currently very important in Asian fishery and aquaculture due to the substantial decline in natural populations because of overexploitation. A large degree of chromosomal variation has been found in this family, mainly through the use of conventional cytogenetic investigations. In this study, we analyzed the karyotype structure and the distribution of 7 repetitive DNA sequences in several *Channa* species from different Thailand river basins. The aim of this study was to investigate the chromosomal differentiation among species and populations to improve upon the knowledge of its biodiversity and evolutionary history. Rearrangements, such as pericentric inversions, fusions and polyploidization, appear to be important events during the karyotypic evolution of this genus, resulting in the chromosomal diversity observed among the distinct species and even among populations of the same species. In addition, such variability is also increased by the genomic dynamism of repetitive elements, particularly by the differential distribution and accumulation of rDNA sequences on chromosomes. This marked diversity is likely linked to the lifestyle of the snakehead fishes and their population fragmentation, as already identified for other fish species. The karyotypic features highlight the biodiversity of the channid fishes and justify a taxonomic revision of the genus *Channa*, as well as of the Channidae family as a whole, as some nominal species may actually constitute species complexes.

## Introduction

The Channidae family (Actinopterygii, Perciformes) comprises 2 genera (*Channa* and *Parachanna*) and 29 recognized species [1]. Although *Channa* contains 26 exclusively Asian species, the extant 3 species of *Parachanna* are African. Molecular phylogenetic analysis estimated that both genera diverged between 40–49 Mya, whereas the Channidae family diverged from other Perciformes approximately 54 Mya [2].

Channids are commonly referred to as “snakeheads” due to the presence of large scales on the heads of most species. These fishes are very important in fishery and aquaculture, and over the years, their wild populations have undergone a substantial decline primarily due to overexploitation [3]. Snakeheads are highly evolved air-breathing fishes capable of surviving for months out of the water when buried in moist soil and are able to migrate over land using wriggling movements [4,5]. These traits imply that dispersal and speciation within the family may not necessarily reflect the processes commonly implied for other obligate freshwater fishes [6,7].

Additionally, such behavior also contributes to the large chromosomal variation found in this family. The diploid number varies from 2n = 32 in *C*. *punctata* to 2n = 112 in *C*. *gachua* [8,9,10], in which Robertsonian rearrangements, pericentric inversions and polyploidy appear to be the main sources of such chromosomal diversity [11,12]. However, cytogenetic studies in this family are still quite scarce. With a few exceptions [12], most studies on the Channidae family used classical cytogenetics data to determine the diploid number and karyotype composition of the species. Therefore, very little is known about other important cytogenetic features, such as the incidence of chromosomal repetitive DNA elements and their evolutionary role in this fish group.

The molecular organization and cytogenetic mapping of repetitive DNA elements, including satellites, multigene families and microsatellite repeats, have been analyzed in a large number of species, demonstrating their enormous potential for expanding the knowledge of the karyotype differentiation in fishes, as reviewed in [13]. In fact, the correlation between repetitive sequences and chromosomal rearrangements has been widely demonstrated [14] because their accumulation in specific genomic areas may induce chromosome breakages, deletions, inversions and amplifications [15,16].

Thus, considering that the high karyotype diversity in Channidae appears to be correlated with speciation events and repetitive DNAs have proven highly effective in showing genomic differentiations, we analyzed the karyotype structure and the distribution of repetitive DNA elements in four *Channa* species (*C*. *lucius*, *C*. *micropeltes*, *C*. *striata* and *C*. *gachua)*. We aimed to investigate chromosomal divergence and probable relationships among species and populations to improve upon the knowledge of the biodiversity and evolutionary events of this important fish family.

## Material and Methods

### Materials

**Fig 1:**
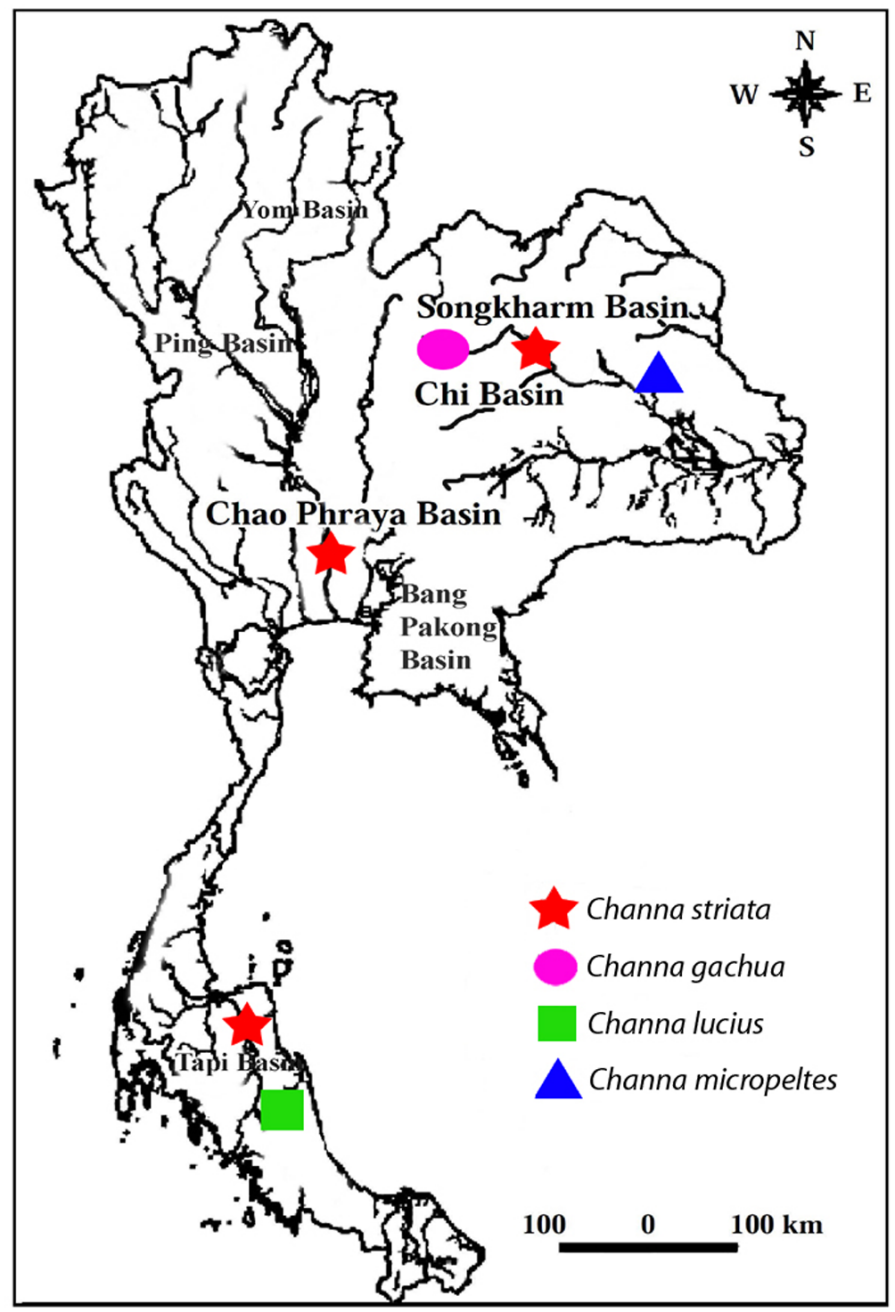
Collection sites of *Channa* species analyzed from Thailand.

Individuals from both sexes of four *Channa* species, *C*. *lucius*, *C*. *micropeltes*, *C*. *striata* and *C*. *gachua*, were analyzed (Table 1). Fishes were collected from different river basins of Thailand (Fig 1). The specimens were caught using hand-net. After capture, animals were placed in sealed plastic bags containing oxygen and clean water and transported to the research station. The experiments followed ethical protocols and anesthesia with clove oil was administered prior to sacrificing the animals to minimize suffering. The process was approved by the Ethics committee of Khon Kaen University and by the RGJ committee under no. PHD/K0081/2556". Mitotic chromosomes were obtained from cell suspensions of the anterior kidney using the conventional air-drying method [17]. The specimens were deposited in the fish collection of the Cytogenetic Laboratory, Department of Biology Faculty of Science, Khon Kaen University.

**Table 1:** Collection sites of the analyzed species with the sample sizes.

Species		N
*Channa striata*	- Chi Basin	(03 ♀; 04 ♂)
*Channa striata*	- Chao Phraya Basin	(06 ♀; 08 ♂)
*Channa striata*	- Tapi Basin	(08 ♀; 07 ♂)
*Channa micropeltes*	- Chi Basin	(12 ♀; 11 ♂)
*Channa lucius*	- Tapi Basin	(08 ♀; 08 ♂)
*Channa gachua*	- Chi Basin	(09 ♀; 07 ♂)

### Chromosome probes and FISH experiments

Two tandemly-arrayed DNA sequences isolated from the genome of an Erythrinidae fish species, *Hoplias malabaricus*, were used as probes. The first probe contained a 5S rDNA repeat copy and included 120 base pairs (bp) of the 5S rRNA transcribed gene and 200 bp of the non-transcribed spacer (NTS) sequence [18]. The second probe corresponded to a 1,400 bp segment of the 18S rRNA gene obtained via PCR from nuclear DNA [19]. The 5S and 18S rDNA probes were cloned into plasmid vectors and propagated in DH5α *Escherichia coli* competent cells (Invitrogen, San Diego, CA, USA).

The 5S and 18S rDNA probes were labeled with Spectrum Green-dUTP and Cy5-dUTP, respectively, using nick translation according to the manufacturer’s recommendations (Roche, Mannheim, Germany).

The microsatellites (CA)_15_, (GA)_15_, (GC)_15_, (CGG)_10_ and (CAA)_10_ were used as probes and were synthesized according to previous work [20]. These sequences were directly labeled with Cy3 at the 5’ terminus during synthesis by Sigma (St. Louis, MO, USA).

Fluorescence *in situ* hybridization (FISH) was performed under high stringency conditions on mitotic chromosome spreads [21]. Metaphase chromosome slides were incubated with RNAse (40 μg/ml) for 1.5 h at 37°C. After denaturation of the chromosomal DNA in 70% formamide/2x SSC at 70°C for 4 minutes, the hybridization mixture (2.5 ng/μl probes, 2 μg/μl salmon sperm DNA, 50% deionized formamide, 10% dextran sulphate) was dropped on the slides, and the hybridization was performed overnight at 37°C in a moist chamber containing 2x SSC. The first post-hybridization wash was performed with 2x SSC for 5 min at 65°C, and a final wash was performed at room temperature in 1x SSC for 5 min. Finally, the slides were counterstained with DAPI and mounted in an antifade solution (Vectashield from Vector Laboratories).

### Image processing

Approximately 30 metaphase spreads were analyzed to confirm the diploid chromosome number, karyotype structure and FISH results. Images were captured using an Olympus BX50 microscope (Olympus Corporation, Ishikawa, Japan) with CoolSNAP and the Image Pro Plus 4.1 software (Media Cybernetics, Silver Spring, MD, USA). Chromosomes were classified as metacentric (m), submetacentric (sm), subtelocentric (st) or acrocentric (a) according to their arm ratios [22].

## Results

### Karyotypes

No differences between male and female karyotypes were observed in all species. Due to the small chromosomal size and the resulting difficulty in precisely distinguishing between m, sm and st chromosomes, all were referred to as bi-armed chromosomes, whereas the acrocentric chromosomes were termed mono-armed chromosomes.

**Fig 2:**
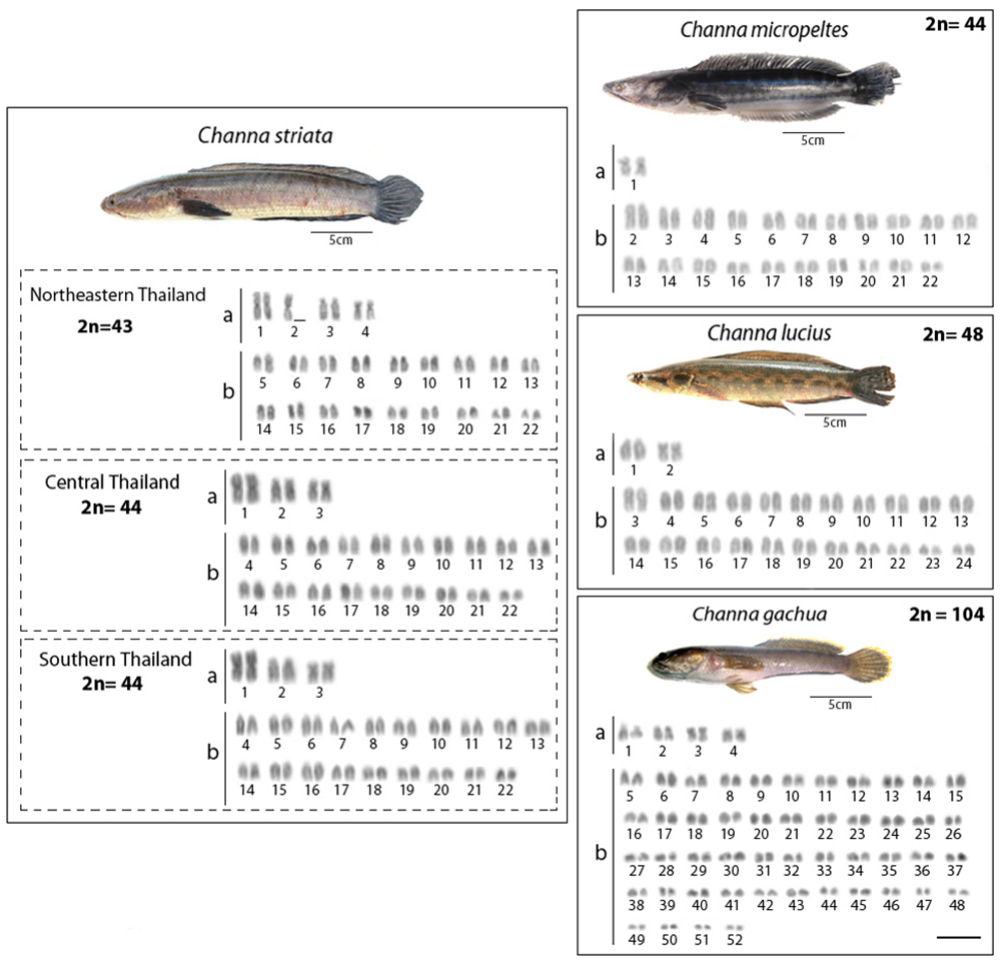
Fish images and karyotypes of Channidae fishes after Giemsa staining. a = bi-armed (m, sm and st) chromosomes and b = mono-armed (a) chromosomes. Scale bars = 5 μm.

*C*. *micropeltes* presented a 2n = 44 (2 bi-armed + 42 mono-armed chromosomes), with the consequent number of chromosome arms (NF) equal to 46. *C*. *lucius* presented a 2n = 48 (4 bi-armed + 44 mono-armed chromosomes) with NF = 52, and *C*. *gachua* presented a 2n = 104 (8 bi-armed + 96 mono-armed chromosomes) with NF = 112 (Fig 2).

For *C*. *striata*, samples of three populations (from southern, central and northeastern Thailand) were analyzed. Both specimens from the southern and central populations presented a 2n = 44 (6 bi-armed + 38 acrocentric chromosomes) with NF = 50. However, all the individuals from the north-northeastern population presented the atypical diploid number of 2n = 43 (7 bi-armed + 36 acrocentric chromosomes) with NF = 50 (Fig 2).

### Chromosome mapping of 5S and 18S rDNA sequences

**Fig 3:**
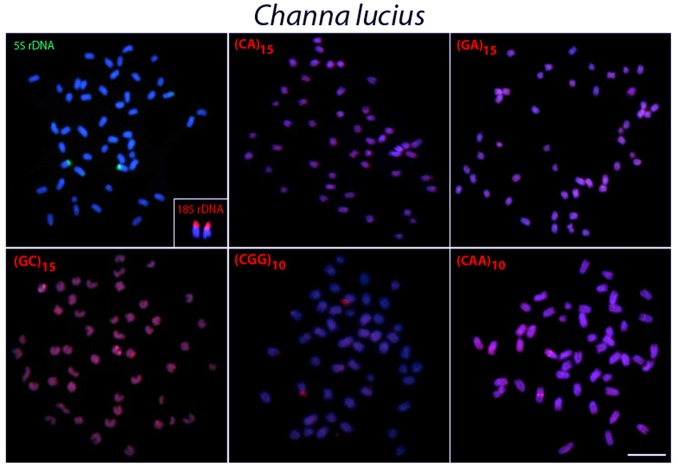
Metaphase plates of *Channa lucius* mapped with different repeated DNAs. 5S rDNA (green), 18S rDNA (red) and di- and trinucleotide microsatellites (red) as probes. The chromosomes with 18S rDNA sites are showned in enlarged forms in boxes. Scale bars = 5 μm.

**Fig 4:**
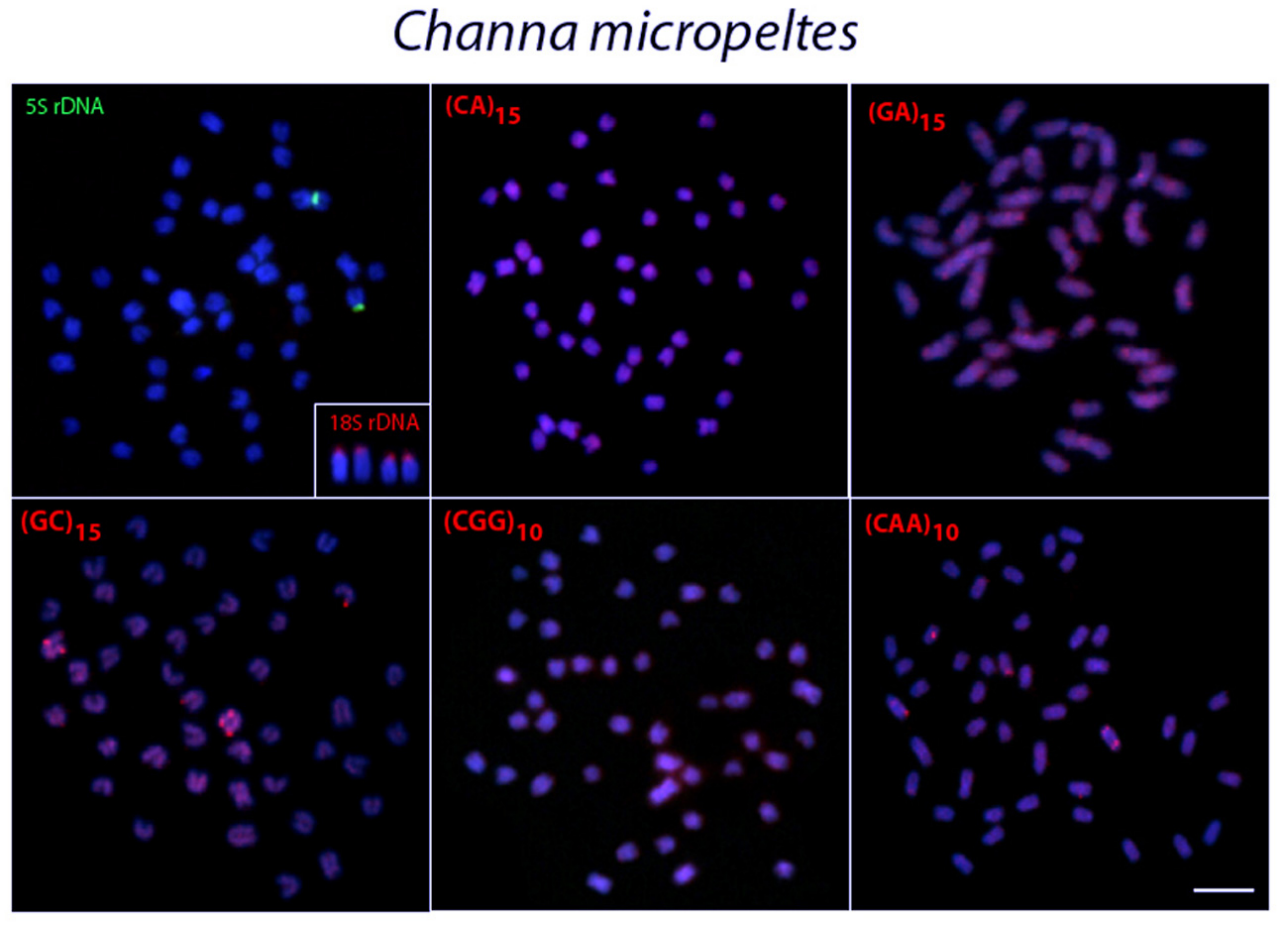
Metaphase plates of *Channa micropeltes* mapped with different repeated DNAs. 5S rDNA (green), 18S rDNA (red) and di- and trinucleotide microsatellites (red) as probes. The chromosomes with 18S rDNA sites are showned in enlarged forms in boxes. Scale bars = 5 μm.

**Fig 5:**
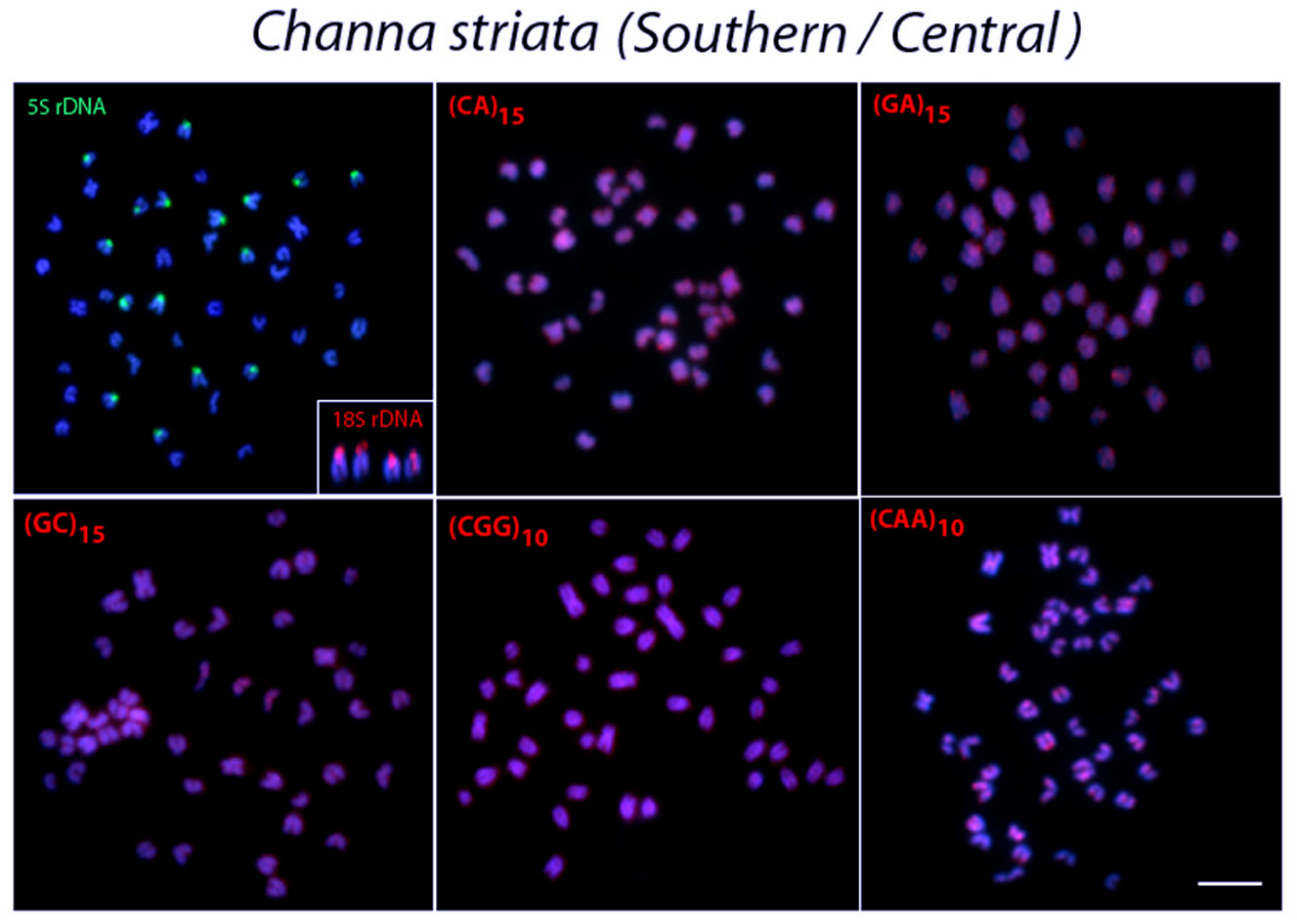
Metaphase plates of *Channa striata* from southern and central Thailand populations mapped with different repeated DNAs. 5S rDNA (green), 18S rDNA (red) and di- and trinucleotide microsatellites (red) as probes. The chromosomes with 18S rDNA sites are showned in enlarged forms in boxes. Scale bars = 5 μm.

**Fig 6:**
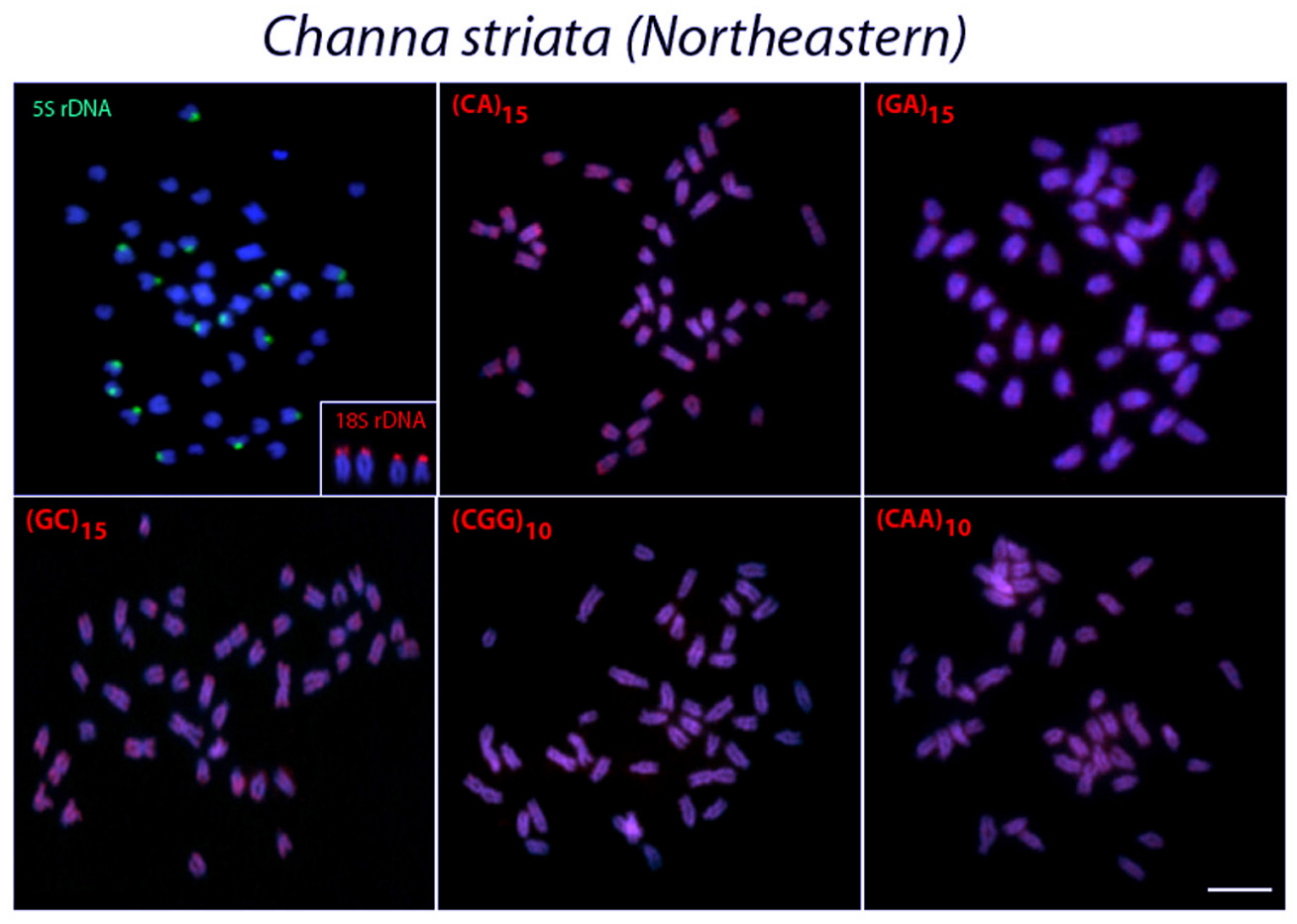
Metaphase plates of *Channa striata* from northeastern Thailand population mapped with different repeated DNAs. 5S rDNA (green), 18S rDNA (red) and di- and trinucleotide microsatellites (red) as probes. The chromosomes with 18S rDNA sites are showned in enlarged forms in boxes. Scale bars = 5 μm.

**Fig 7:**
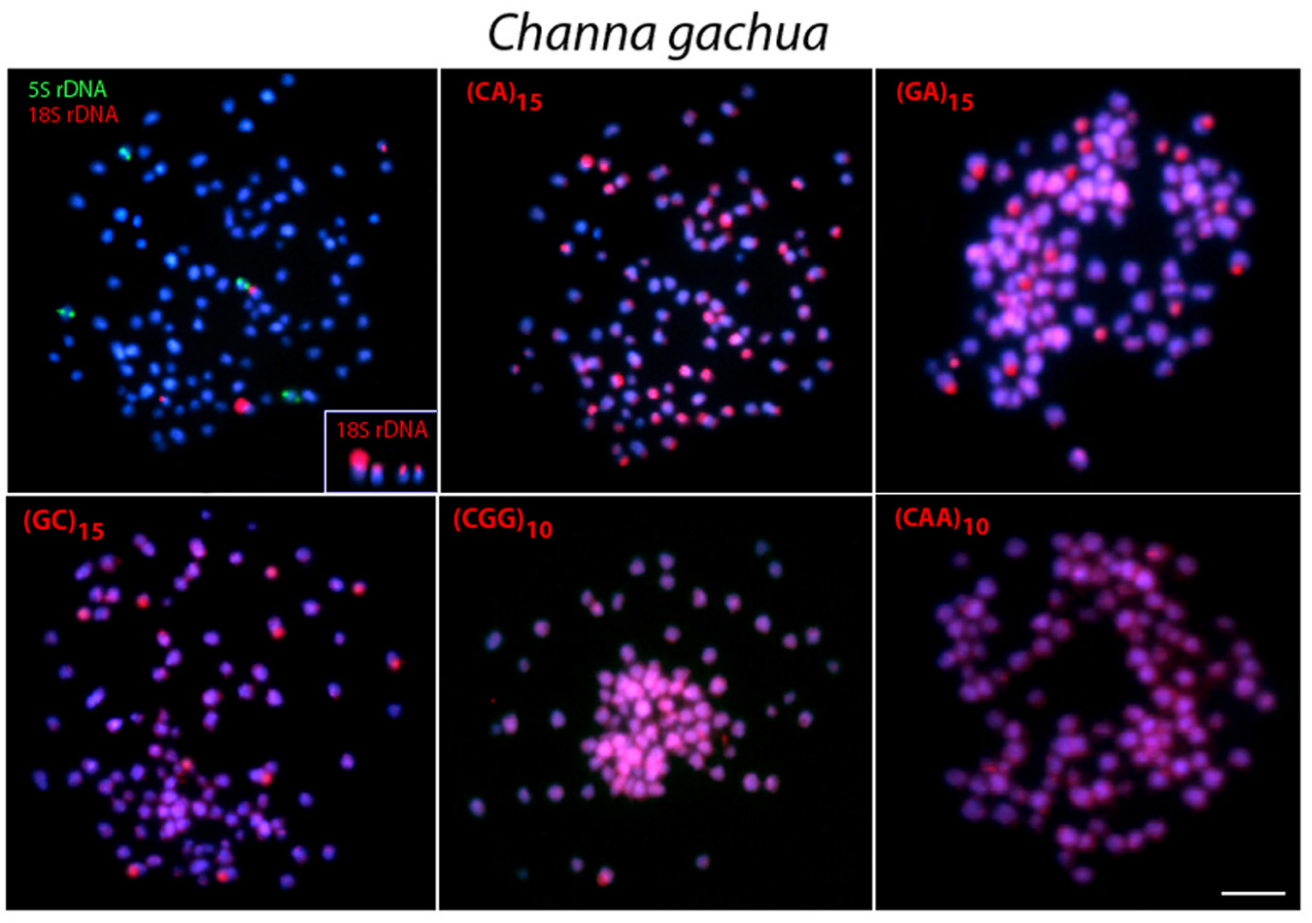
Metaphase plates of *Channa gachua* mapped with different repeated DNAs. 5S rDNA (green), 18S rDNA (red) and di- and trinucleotide microsatellites (red) as probes. The chromosomes with 18S rDNA sites are showned in enlarged forms in boxes. Scale bars = 5 μm.

The probe for the 18S rDNA hybridized to the subtelomeric/telomeric region of two chromosome pairs in all species analyzed, except for *C*. *lucius*, in which only one pair was labeled with this probe (Figs 3–7, in the detail).

In contrast, 5S rDNA sequences showed a widely distinct distribution among species, although they were always located in the centromeric region of the chromosomes. Thus, 5S rDNA sequences were located in only one chromosome pair in *C*. *micropeltes* and *C*. *lucius* (Fig 4) but were present in two chromosome pairs in *C*. *gachua* (Fig 7). In turn, all 3 populations of *C*. *striata* showed a surprising increase in the number of 5S rDNA sites, with 8 chromosomal pairs bearing these sequences (Figs 5 and 6).

### Chromosome Mapping of Microsatellite Sequences

In *C*. *micropeltes*, *C*. *lucius* and in the three populations of *C*. *striata*, most microsatellites were abundantly distributed in all chromosomes with the exception of (CG)_15_, (CGG)_10_ and (CAA)_10_, which showed stronger hybridization signals on two chromosomes in *C*. *lucius*, and (GC)_15_ and (CAA)_10_, which also displayed several more conspicuous sites in *C*. *micropeltes* (Figs 3–6). However, in *C*. *gachua*, the three dinucleotide microsatellites, (CA)_15_, (GA)_15_ and (GC)_15_, were highly accumulated in several chromosomes (Fig 7).

## Discussion

### Chromosomal evolution among *Channa* species

Morphological diversification among most Perciformes fishes is not accompanied by a conspicuous karyotype diversification [23]. However, this scenario does not appear to be the case for the Channidae family, in which a remarkable chromosomal diversity is observed.

The Thailand region contains seven recognized species of the genus *Channa*, with cytogenetic data known for six of them [24]. The evolutionary relationships for Asian snakeheads fishes and their probable divergence times within the Channidae family were proposed by one study [2] based on phylogenetic analyses. In addition, a putative evolutionary pathway based on the karyotypes of some *Channa* species highlighted a parallel with Adamson’s data [25]. Among these species, *C*. *lucius* occupies the most basal position and is closely related to *C*. *diplogramme* and *C*. *micropeltes*. In fact, these species are characterized by a region of gular scales that is also present in the African sister group *Parachanna* but absent in the majority of other Asian species [26]. Accordingly, *C*. *lucius* possesses 2n = 48 chromosomes, which was proposed as the basal number for Perciformes [27]. The decrease in the diploid number in the other Channa species analyzed (with the exception of *C*. *gachua*), with concomitant changes in the number of the bi-armed chromosomes, suggests that fusions and pericentric inversions were the main chromosomal rearrangements related to the karyotypic evolution of this genus. In fact, such rearrangements have been frequently detected in several species of Perciformes and are considered to be effective tools to characterize derived karyotypes [28]. Indeed, it is likely that major changes in karyotype structure may lead to a reorganization of co-adapted gene complexes; for this reason, these changes are an important evolutionary source [29,30].

Karyotype diversification processes and morphological patterns are often indicators of the lifestyle of a species [31,32]. Although Channidae species have a wide geographical distribution, their low vagility facilitates the maintenance of chromosomal rearrangements in small populations and, consequently, an increase in their chromosomal diversity. This same scenario is also found with the Neotropical fish *Hoplias malabaricus* (Erythrinidae), which represents a “species complex” with a wide geographic distribution and a low vagility. This fish is outstanding in its karyotypic diversity, with well-defined population differences in diploid numbers and chromosomal morphology and distinct sex chromosome systems [33,34].

The *in situ* investigation of repetitive DNA elements provided useful characteristics for comparative genomics at the chromosomal level, offering new insights into the karyotype evolution of *Channa* species. The number and distribution of ribosomal genes were not conserved among species, although the distribution of 18S rDNA sequences was less variable than that of 5S rDNA. All species presented four 18S rDNA sites, except *C*. *lucius*, in which only two incidences of this type of sequence were found (Figs 2–7). In contrast, the distribution pattern of the 5S rDNA sites showed a large variation. Thus, whereas 2 or 4 sites were mapped on the chromosomes of *C*. *lucius*, *C*. *micropeltes* and *C*. *gachua*, a total of 16 sites were observed in all *C*. *striata* populations analyzed (Figs 2–7). In this sense, *C*. *striata* is thought to be the most differentiated species, considering the 5S rDNA distribution in the karyotype. Coincidentally, these data support the chromosomal phylogeny proposed by one report [25], where *C*. *striata* was located in a different evolutionary path than that shared by *C*. *lucius*, *C*. *micropeltes* and *C*. *gachua*.

Hypervariability in number and location of rDNA loci was previously described for several fish groups [35–38]. In the red wolf fish, *Erythrinus erythrinus*, it was demonstrated that chromosomal rearrangements and genomic modifications due to rDNA spreading were significant events during the evolutionary history of this fish [35,36]. Additionally, the synteny of 5S rDNA genes and a retrotransposable element also indicated that chromosomes may have undergone rearrangements mediated by retrotransposon activity during evolution [35]. However, at the moment, we have no information on the possible role of transposable elements in increasing the number of 5S rDNA sites in *C*. *striata*, which will be the goal of further investigations. In fact, the particular incidence of such elements in the genome of this species will be a useful tool to strengthen this hypothesis.

Microsatellites, which are abundant repeated sequences that are present in all eukaryotic genomes studied thus far, are found either between the coding regions of structural genes or between other repetitive sequences [39]. In fish species such as *H*. *malabaricus*, *Triportheus trifurcatus*, *Oplegnathus fasciatus* and *Leporinus elongatus*, microsatellite repeats show a conspicuous accumulation in the telomeric regions of chromosomes [40–43]. However, only a discrete banding pattern was observed for some microsatellites in the *Channa* species analyzed, with a small accumulation of (GC)_15_, and (CAA)_10_ in specific chromosomal pairs in *C*. *micropeltes* and *C*. *lucius*, with this later also presenting also a (CGG)_10_ accumulation (Figs 3 and 4). However, *C*. *gachua* represented an exception to this general scenario, since a strong accumulation of some repeats was presented in several chromosomes (Fig 7). As this species has a polyploid origin [25], this differential accumulation of repetitive sequences may be due to its particular evolutionary history, as discussed below.

### Chromosomal variability among *C*. *striata* populations

A comparative population analysis showed that *C*. *striata* differs in chromosome number and karyotype structure. Thus, although populations from southern and central Thailand presented 2n = 44 chromosomes, all the individuals from the northeastern population presented an atypical 2n = 43 chromosomes (Fig 1) and were distinguished by the presence of one particularly large m chromosome. Accordingly, some previous studies have also demonstrated that *C*. *striata* from Thailand contains a conspicuous karyotypic variation, with diploid numbers ranging from 2n = 40 to 2n = 44 [24,25].

In the multi-locus molecular phylogeny proposed by [2], the same *C*. *striata* populations were sampled; whereas all other *Channa* species formed a single monophyletic group, the divergence among *C*. *striata* populations was the highest. At the intra-specific level, *C*. *striata* populations were separated into three distinct and highly divergent clades. In accordance with the cytogenetic data, populations from central and southern Thailand were placed in a single group, whereas populations from northeastern Thailand comprised a divergent group [2].

Previous analysis of *C*. *striata* from the same northeastern Thailand region showed a karyotype structure of 2n = 42 chromosomes, with 8 bi-armed + 34 mono-armed chromosomes, i.e., with a reduction in the diploid number and the addition of two large m chromosomes compared with individuals from central and southern populations, which is likely due to centric fusion rearrangements [24]. In view of these findings, it is possible that *C*. *striata* with 2n = 43 chromosomes may correspond to a hybrid form from parental specimens with 2n = 42 x 2n = 44 chromosomes or, alternatively, to specimens heterozygotic for the centric fusion that gave origin to the 2n = 42 karyotype.

One remarkable feature present in all populations analyzed was the high number of 5S rDNA sites. Several adaptive conditions with significant evolutionary relevance may be associated with the rapid dispersion of specific sequences over a short time period [44]. For example, in the sister species *Coregonus albula* and *C*. *fontanae*, the spreading of rDNA sites affected the recombination rates in both genomes and led to a rapid genomic divergence, resulting in a faster ecological speciation event [37]. *C*. *striata* is currently the most common snakehead fish found in southern Asia and is largely exploited in the fish market due to its large natural distribution that includes many freshwater habitats. Thus, as well as in the *Coregonus* species, a rapid genome differentiation could help intensify divergences among distinct populations of *C*. *striata*. In fact, the sum of the available data for *C*. *striata* shows a clear chromosomal differentiation in this important fish group, which may correspond to a possible species complex.

### The polyploidy status of *C*. *gachua*

The chromosomal analysis indicates that *C*. *gachua* corresponds to a polyploid species within the genus *Channa* with 4n = 104 chromosomes. However, this is not the only known case of polyploidy in this genus, as *C*. *stewartii* represents an autotetraploid species [11]. Although polyploidy represents an uncommon trait in higher vertebrates, it occurs independently and often repeatedly in many fish species [45]. The advantage of polyploidy in fishes is not only an increase in their potential for adaptability to the environment but also an enhancement of their reproductive efficiency [45]. In fact, *C*. *gachua* can live in higher mountain areas with fluctuating climates and can tolerate very stagnant, poorly oxygenated, turbid and even foul water, besides being the dominant species in the habitat [10,46].

A high degree of karyotype diversity has been reported for *C*. *gachua*, with the diploid number ranging from 78 to 112 chromosomes [10]. One of these populations, with 2n = 52 chromosomes, may likely be linked to the origin of the current analyzed polyploid individuals. Thus, as considered for *C*. *striata*, the karyotypic data for *C*. *gachua* corroborate the view that distinct populations of this species cannot be considered an evolutionary unit and likely resemble an assemblage of species with unresolved taxonomy.

The repetitive DNA fraction of the genome plays an important role during polyploidization and post-polyploidization changes [47,48]. After polyploidy, an eventual diploidization process may occur that leads to the loss of a subset of genes and the accumulation of repetitive DNA elements in many genomic areas. Because these sequences evolve faster than unique sequences and genes, they tend to colonize these new “ghost towns” and rapidly accumulate on the chromosomes [37,49]. Additionally, a parallel can also be traced to the evolution of the sex chromosomes, in which the “gene deserts” found in the sex-specific chromosomes (Y or W) serve as ideal niches for the long-term survival of these elements due to the weaker selection in these regions [50,51]. Similar events could also explain the strong accumulation of repeated DNA elements that was found in several chromosomes of *C*. *gachua* compared with the other diploid *Channa* species analyzed.

However, a repetitive DNA complex can also mediate ectopic chromosomal exchanges when homologous and (or) non-homologous chromosome recombination moves sequences within and between genomes. Furthermore, insertions of repeated DNA elements may create a new crossing-over ‘hot spot’ that promotes homologous or non-homologous chromosome rearrangements. The latter include spontaneous translocations, inversions and deletions and are potential mechanisms for rapid genome reorganization during speciation and stabilization of polyploids [14]. Therefore, whether the accumulation of repetitive DNA elements is the cause or the consequence of the cytogenetic events responsible for the emergence of polyploidy in *C*. *gachua* still requires further investigation.

## Conclusions

Channidae fishes present a remarkable evolutionary dynamism. In the genus *Channa*, this attribute is mediated by different chromosomal rearrangements, such as pericentric inversions, fusions and polyploidization, resulting in a high karyotypic variation among species and populations. Additionally, such variability is also reinforced by the dynamism of repetitive elements in the genome, especially by the differential distribution and accumulation of rDNA sequences among chromosomes. Although not yet completely understood, this marked diversity is likely linked to the lifestyle of these fishes and to population fragmentation, as already identified for other fish species. In turn, these karyotypic features justify a taxonomic revision of the genus *Channa*, and of the Channidae family as a whole, in light of the observation that some nominal species may actually constitute species complexes.
